# Relationship Between Anti-Müllerian Hormone and *In Vitro* Fertilization-Embryo Transfer in Clinical Pregnancy

**DOI:** 10.3389/fendo.2020.595448

**Published:** 2020-12-04

**Authors:** Xing Yu Sun, Yun Zhu Lan, Shuang Liu, Xiao Ping Long, Xi Guang Mao, Ling Liu

**Affiliations:** ^1^ Department of Obstetrics and Gynecology, The Affiliated Hospital of Southwest Medical University, Luzhou, China; ^2^ Department of Reproductive Center, The Affiliated Hospital of Southwest Medical University, Luzhou, China; ^3^ Department of Obstetrics and Gynecology, Affiliated Traditional Chinese Medicine Hospital, Southwest Medical University, Luzhou, China

**Keywords:** anti-Müllerian hormone, pregnancy, *in vitro* fertilization-embryo transfer, assisted reproductive technologies, infertile women

## Abstract

**Objectives:**

To retrospectively analyze the correlation between anti-Müllerian hormone (AMH) and the number of oocytes obtained by controlled ovarian hyperstimulation (COH) in women of different ages and explore the factors affecting *in vitro* fertilization and embryo transfer (IVF-ET) in clinical pregnancy of infertile women to provide evidence for infertile women to choose assisted reproduction strategies.

**Methods:**

Infertile women who received IVF-ET or intracytoplasmic sperm injection and embryo transfer (ICSI-ET) treatment in the reproductive center of XX hospital between October 2018 and September 2019 were included. Patient data on medical records, age, body mass index (BMI), years of infertility, basic follicle-stimulating hormone (FSH), basic luteinizing hormone (LH), basic estradiol (E_2_), anti-Müllerian hormone level (AMH), antral follicle count (AFC), gonadotropins (Gn) medication days, Gn dosage, endometrial thickness on transplantation day, the number of retrieved oocytes, the number of mature oocytes obtained, the number of embryos transferred, clinical pregnancy status, etc., were collected.

**Results:**

A total of 314 patients were enrolled in this study, with an average age of 31.0 ± 4.5 years. The infertility period ranged from 0–21 years. The AMH level showed a downward trend with increasing age. Overall, the AMH level of women of all ages was positively correlated with the number of retrieved oocytes (*r* = 0.335, *p* < 0.001). The AMH level of women between 22 and 28 years old was positively correlated with the number of retrieved oocytes (*r* = 0.164, *p* < 0.061) but it was not statistically significant. Similarly, the AMH level of women aged 29–35 and 36–43 was positively correlated with the number of retrieved oocytes (*r* = 0.356, *p* < 0.001; *r* = 0.461, *p* < 0.001). The average age of the pregnant group (30.6 ± 4.4 years) was lower than that of the non-pregnant group (32.2 ± 4.6 years) (*p* < 0.001). The number of oocytes obtained (9.8 ± 4.5) and the number of embryos transferred (1.9 ± 0.4) in the pregnant group was significantly higher than that in the non-pregnant group (9.2 ± 4.5; 1.7 ± 0.5); the difference was statistically significant. The multivariate logistic regression model showed that age (OR = 0.574 95% CI: 0.350–0.940), AMH (OR = 1.430 95% CI: 1.130–1.820) and the number of oocytes obtained (OR = 1.360 95% CI: 1.030–1.790) were factors affecting clinical pregnancy.

**Conclusion:**

We found that the level of AMH in infertile women decreased with age and the number of oocytes obtained in infertile women was positively correlated with AMH. Moreover, the number of oocytes and embryo transferred in the pregnant group was significantly higher than those in the non-pregnant group. Furthermore, age, AMH and the number of oocytes affected the clinical pregnancy.

## Introduction

In recent years, the fertility problem of infertile women has become a major challenge for reproductive doctors. Assisted reproductive technologies (ARTs) are an important alternative to solve the problem of infertile women. However, the success of pregnancy depends on various factors, including gamete quality, uterine receptivity and immune factors. Among them, the key factors affecting infertile women are the decrease in the number and quality of oocytes.

At present, indicators for evaluating ovarian reserve function include age, follicle-stimulating hormone (FSH), anti-Müllerian hormone (AMH), and antral follicle count (AFC). Among them, AMH is a member of the transforming growth factor β (TGF-*β*) superfamily. It is a dimer glycoprotein formed by disulfide bonds. In women, antral follicles and small follicles secrete AMH, which has the effect of promoting growth and differentiation. The level of AMH is positively correlated with the number of antral follicles and small follicles in the ovary. Studies have shown that AMH can not only reflect the number of antral follicles but also the quality of oocytes (1, 2). Generally, it is believed that AMH is maintained throughout the menstrual cycle and it is stable (3–5). Therefore, AMH is considered to be the best indicator to evaluate ovarian reserve. The role of AMH in ART has always been one of the research hotspots in the field of assisted reproduction. Controlled ovarian hyperstimulation (COH) is a key part of assisted reproductive technology and plays a vital role in the success or failure of treatment. Several clinical studies have shown that patients with low AMH levels have a higher cancellation rate of oocytes retrieval cycles because of low ovarian response (6–9). Some researchers also pointed out that AMH levels are correlated with the number of oocytes retrieved, fertilization rate, number of embryos available and clinical pregnancy (10–12). However, the relationship between AMH and the number of oocytes retrieved in infertile women at different ages is yet to be studied.

Herein, we analyze the correlation between AMH and the number of retrieved oocytes obtained by COH in women of different ages and explores the factors affecting *in vitro* fertilization and embryo transfer (IVF-ET) in clinical pregnancy of infertile women to provide a basis for infertile women to choosing assisted reproduction strategies.

## Methods

### Patient Enrollment and Data Collection

This is a retrospective study of infertile women who received IVF-ET or intracytoplasmic sperm injection and embryo transfer ICSI-ET treatment in the reproductive center of XX hospital from October 2018 and September 2019. Estimate sample size based on “multi-stage random sampling survey”. The sample size was estimated to obtain the prevalence within 1 of the true value at 1 level of significance. This kind of sample design has an impact, called design effect on sampling variability. As a result of this impact, the obtained sample size was multiplied by 1.5 and the outcome was taken as the final sample size. Patients who (1) underwent IVF-ET or ICSI-ET treatment; (2) promoted ovulation through long, short or antagonist programs; and (3) had bilateral ovaries based on transvaginal color Doppler ultrasound were included. Patients with (1) metabolic abnormalities or endocrine diseases (such as polycystic ovary syndrome, diabetes, abnormal thyroid function, Cushing syndrome, etc.); (2) chromosomal abnormalities in either spouse; (3) a history of ovarian surgery; (4) infectious diseases, autoimmune diseases, allergic diseases, tumors, hepatitis and other diseases; (5) a history of thrombosis or family history; and (6) endometriosis were excluded.

Patient data on medical records, age, body mass index (BMI), years of infertility, basic FSH, basic luteinizing hormone (LH), basic estradiol (E_2_), AMH level, AFC, gonadotropins (Gn) medication days, Gn dosage, endometrial thickness (mm) on transplantation day, the number of oocytes obtained, the number of mature oocytes obtained, the number of embryos transferred and clinical pregnancy status etc. were collected.

### Clinical Indicators

#### AMH Test

The blood samples of all patients were stored at -20°C and tested by an enzyme-linked immunoassay kit (ELISA, AMH quantitative test kit, Guangzhou Kangrun Biotechnology Co., Ltd.). The minimum detectable AMH concentration was 0.06 ng/ml, the relevant coefficient (r) ≥0.9900, the relative deviation of the measurement results was within 10% and the coefficient of variation (CV) ≤ 10%.

#### Judgment of Oocyte Maturity

According to the development and morphological evaluation of the oocyte, the oocyte is divided into three classes, mature oocyte (MII stage): the endogenous foaming of the oocyte cytoplasm disappears and the first polar body can be seen in the perivitelline space; the intermediate mature oocyte (MI phase): the endogenous foaming of the oocyte cytoplasm disappears and the perivitelline space is not seen first in the polar body; immature oocytes (GV stage): germinal follicles can be seen in the cytoplasm. Based on the oocyte maturity criterion, the number of mature oocytes obtained in this study was that obtained at the MII stage (13).

#### Embryo Quality Judgment

According to embryo development speed and morphological score, embryos are divided into 4 Grades, Grade I: uniform blastomere size, homogeneous and transparent cytoplasm, no debris; Grade II: uniform blastomere size, with debris <20%; Grade III: unequal size of blastomeres, with more fragments (20–50%); Grade IV: unequal size of blastomeres, fragments >50%. Based on these embryo quality determination standards, Grades I and II embryos were high-quality embryos in our laboratory.

Rate of retrieved mature oocytes=number of retrieved mature oocytes/number of retrieved oocytes(%)

Clinical pregnancy rate=number of clinical pregnancy cycles/number of hyperstimulation cycles (%)

### Statistical Analysis

All data were reported as number and percentage, while continuous data were reported as mean ± standard deviation (SD). We used the chi-square (χ^2^) test or the Fisher’s exact test to compare categorical data. Mann-Whitney-Wilcoxon test was applied to compare nonnormally continuous variables. Pearson correlation and multiple linear regression were used to analyze the correlation between AMH level and the number of oocytes obtained. Logistic regression models were used to evaluate the influencing factors of clinical pregnancy. All statistical analyses were performed using SPSS 23.0 (SPSS Inc, Chicago, Ill). A value of *p* < 0.05 was considered statistically significant.

## Results

### Patient Characteristics

A total of 314 patients aged between 22 and 43 years old were selected, with an average age of 31.0 ± 4.5 years old. The infertility period was between 0 and 21 years, with an average age of 4.4 years. Detailed patient characteristics are shown in **Table 1**.

**Table 1:** Baseline clinical parameters of the patients.

Clinical parameters		Patient group (n = 314)
The age of women		31.0 ± 4.5
The age of men		33.1 ± 5.8
Years of infertility		4.4 ± 3.5
BMI(kg/m^2^)		22.6 ± 4.3
Basic FSH(mIU/ml )		9.0 ± 3.4
Basic LH(mIU/ml )		4.2 ± 4.2
Basic E_2_(pg/ml )		182.7 ± 473.7
Basic PRL		14.3 ± 11.2
Basic T		49.5 ± 23.3
Basic P		1.7 ± 4.3
AMH(ng/ml )		5.3 ± 4.3
Intimal thickness (mm)		6.5 ± ± 2.7
Number of eggs obtained (pieces)		9.3 ± 4.5
The total of Gn		2641.0 ± 975.9
The total days of Gn		11.1 ± 2.1
Number of embryos transferred (pieces)		1.7 ± 0.5
Number of MII (pieces)		8.5 ± 4.3
Rate of obtaining mature eggs %		90.7 ± 10
Ending	Clinical pregnancy	104(33.1%)
	Biochemical pregnancy	40(12.7%)
	Not pregnant	162(51.6%)

BMI, body mass index; FSH, follicle stimulating hormone; LH, luteinizing hormone E_2_, estradiol; AMH, Anti-Mullerian hormone; Gn, gonadotropins.

### Comparison of Different Parameters of Patients in Different Age Groups

In this study, the ages of the female patients were divided into three groups: 22–28, 29–35, and 36–43 years old. The duration of infertility varied among the age groups (**Table 2**). The average duration of infertility increased with an increase in age. Women between the ages of 36 and 43 had the longest duration of infertility, with an average of about 5.7 years. The total dosage of basal FSH and Gn increased with an increase in age, while basal LH, basic E_2_, basic prolactin (PRL), basic testosterone (T), AMH, and the number of oocytes harvested and the number of MII retrieved oocytes all showed a decreasing trend. Age (woman and man), infertility years, basic PRL, and AMH showed significant differences in different age groups.

**Table 2:** Baseline data of different age groups of women.

Clinical parameters		22–28 (n = 90)	29–35 (n = 169)	36–43 (n = 55)	*P*
The age of women		25.8 ± 1.9	31.4 ± 1.9	38.1 ± 2.1	<0.001
The age of men		28.2 ± 3.3	33.6 ± 4.3	39.5 ± 6.3	<0.001
Years of infertility		3.3 ± 1.8	4.6 ± 3.2	5.7 ± 5.4	0.001
BMI(kg/m^2^)		22.4 ± 4.1	22.7 ± 3.7	22.5 ± 6.0	0.83
Basic FSH(mIU/ml )		8.5 ± 3.0	9.2 ± 3.7	9.6 ± 2.9	0.294
LH(mIU/ml )		4.4 ± 4.8	4.1 ± 3.6	4.0 ± 5.2	0.736
Basic E_2_ (pg/ml )		228.6 ± 569.8	171.7 ± 425.4	141.4 ± 444.1	0.581
PRL		16.9 ± 17.6	13.4 ± 7.3	12.9 ± 6.2	0.018
Basic T		55.1 ± 25.0	48.7 ± 23.4	42.9 ± 17.7	0.072
Basic P		1.7 ± 3.6	1.8 ± 4.8	1.7 ± 3.7	0.993
AMH(ng/ml )		6.6 ± 4.4	5.2 ± 4.4	3.4 ± 2.8	0.039
Intimal thickness(mm)		6.5 ± 2.5	6.5 ± 2.7	6.4 ± 2.8	0.999
Number of eggs obtained (pieces)		9.9 ± 4.1	9.5 ± 4.9	7.8 ± 3.3	0.795
The total of Gn		2373.2 ± 1002.7	2632.4 ± 933.0	3105.0 ± 903.5	0.071
The total days of Gn		11.3 ± 2.3	11.1 ± 1.9	11.0 ± 2.2	0.48
Number of embryos transferred (pieces)		1.6 ± 0.5	1.7 ± 0.5	1.7 ± 0.6	0.099
Number of MII (pieces)		9.2 ± 4.0	8.5 ± 4.7	7.3 ± 3.4	0.421
Rate of obtaining mature eggs %		92.3 ± 0.1	90.0 ± 0.2	92.6 ± 0.1	0.212
Ending	Clinical pregnancy	30(33.3%)	61(36.1%)	14(25.5%)	0.988
	Biochemical pregnancy	11(12.2%)	21(12.4%)	8(14.5%)	
	Not pregnant	48(53.3%)	83(49.1%)	31(56.4%)	

BMI, body mass index; FSH, follicle stimulating hormone; LH, luteinizing hormone E_2_, estradiol; AMH, Anti-Mullerian hormone; Gn, gonadotropins.

### AMH Levels in Different Age Groups

**Figure 1:**
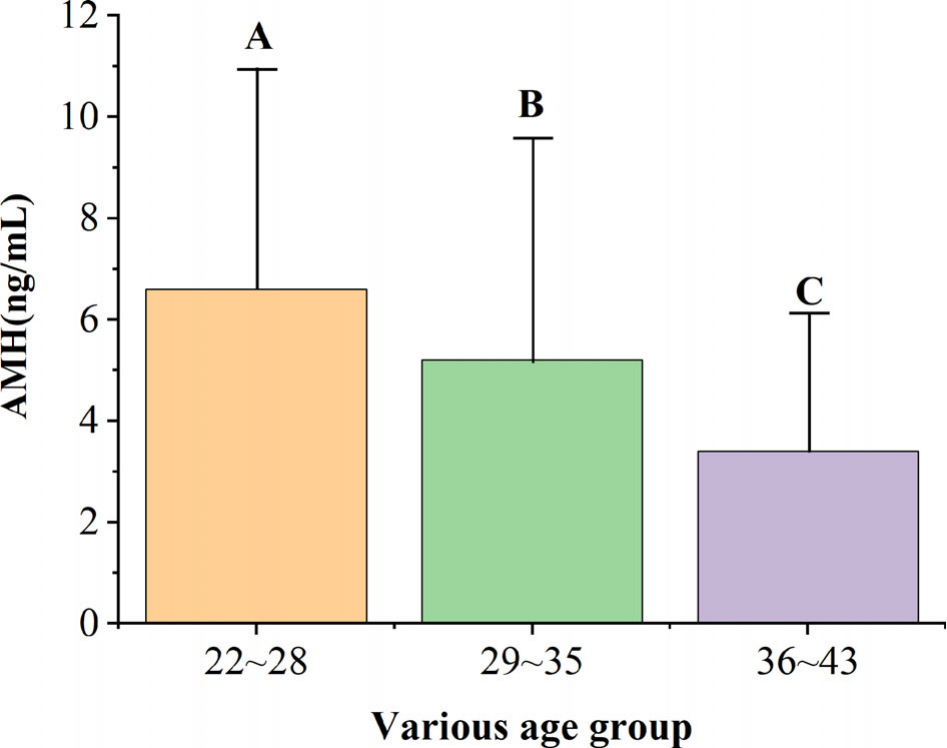
AMH levels in women of different ages.

**Figure 1** shows the average and SD of AMH levels for women of different ages. There was a significant difference in the AMH levels among the three age groups. In addition, AMH levels decreased with increasing age (**Table 3**). AMH levels of women aged 22–28 were significantly higher than those aged 29–35. However, AMH levels of women aged 29–35 were significantly higher than those aged 36–43. Similarly, AMH levels of women aged 22–28 were significantly higher than those aged 36–43.

**Table 3:** AMH levels in women of different ages.

Age (years )	Number	The level of AMH(ng/ml )
22**–**28	90	6.6 ± 4.4^A^
29**–**35	169	5.2 ± 4.4^B^
36**–**43	55	3.4 ± 2.8^C^

AMH, Anti-Mullerian hormone.

### Correlation Between the AMH Level and the Number of Oocytes Obtained

**Figure 2:**
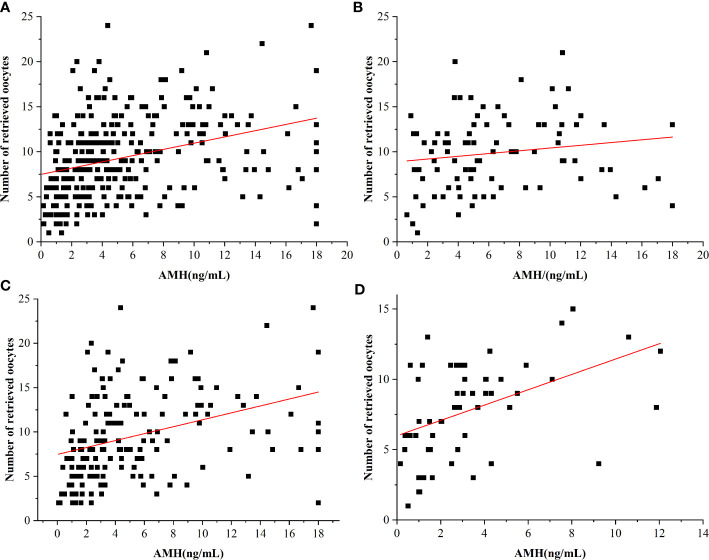
Relationship between AMH levels and the number of retrieved oocytes in different age groups. **(A)** Overall relationship. **(B)** 22–28 years. **(C)** 29–35 years. **(D)** 36–43 years.

**Figure 2** and **Table 4** show the correlation between the AMH level and the number of oocytes obtained in women of different ages. Overall, in women of all ages, the level of AMH was positively correlated with the number of oocytes retrieved (*r* = 0.335, *p* < 0.001; **Figure 2A**). The level of AMH was positively correlated with the number of retrieved oocytes in women aged 22–28 (*r* = 0.164, *p* < 0.061; **Figure 2B**) but not statistically significant due to the small sample size and sampling errors. In addition, the level of AMH was positively correlated with the number of oocytes obtained in women aged 29–35 (*r* = 0.356, *p* < 0.001; **Figure 2C**). Moreover, there was a positive correlation between the level of AMH and the number of oocytes obtained in women aged 36–43 (*r*= 0.461, *p* < 0.001; **Figure 2D**).

**Table 4:** The relationship between AMH and the number of eggs obtained in different age groups.

Age	Linear regression equation	*r*	*P*
Total	y = 0.35x+7.49	0.335	<0.001
22**–**28	y = 0.15x+8.88	0.164	0.061
29**–**35	y = 0.39x+7.46	0.356	<0.001
36**–**43	y = 0.55x+5.99	0.461	<0.001

AMH, Anti-Mullerian hormone.

### Comparison of Factors Influencing IVF-ET Clinical Pregnancy in Infertile Women

Age, number of years of infertility, BMI, basic FSH, basic LH, basic E_2_, basic PRL, basic T, basic progesterone (P), AMH, intimal thickness, number of oocytes harvested, and total Gn dosage of pregnant and non-pregnant patients were compared. The total number of Gn days, the number of embryos transferred, the number of MII oocytes and the rate of mature oocytes were statistically significant in the female age, the number of oocytes obtained and the number of embryos transferred between the two groups. The average age of women in the pregnant group and the non-pregnant group was 30.6 ± 4.4 years and 32.2 ± 4.6 years, respectively. The average age of the pregnant group was lower than that of the non-pregnant group (*p* < 0.001). The number of oocytes obtained and the number of embryos transferred was significantly higher in the pregnant group than in the non-pregnant group and the difference was statistically significant (**Table 5**).

**Table 5:** Comparison of data between pregnant group and non-pregnant group.

Clinical parameters	Non-pregnant group(*n =* 209)	*χ* ^2^/*t*	*P*
The age of women	32.2 ± 4.6	-10.143	<0.001
The age of men	33.3 ± 6.0	-0.748	0.455
Years of infertility	4.7 ± 3.7	-1.878	0.062
BMI(kg/m^2^)	22.4 ± 3.9	0.91	0.364
Basic FSH(mIU/ml )	8.9 ± 3.5	0.687	0.492
Basic LH(mIU/ml )	4.2 ± 4.3	0.034	0.973
Basic E_2_(pg/ml )	181.8 ± 470.4	0.047	0.962
Basic PRL	14.1 ± 9.4	0.561	0.575
Basic T	49.1 ± 23.2	0.451	0.652
Basic P	1.5 ± 3.7	1.148	0.252
AMH(ng/ml )	5.1 ± 4.2	1.078	0.282
Intimal thickness(mm)	6.3 ± 2.6	1.387	0.167
Number of eggs obtained (pieces)	9.2 ± 4.5	6.514	0.001
The total of Gn	2644.5 ± 960.5	-0.094	0.925
The total days of Gn	11.1 ± 2.1	0.736	0.463
Number of embryos transferred (pieces)	1.7 ± 0.5	5.292	0.012
Number of MII (pieces)	8.4 ± 4.4	0.463	0.644
Rate of obtaining mature eggs %	90.8 ± 0.1	-0.304	0.761

BMI, body mass index; FSH, follicle stimulating hormone; LH, luteinizing hormone E_2_, estradiol; AMH, Anti-Mullerian hormone; Gn, gonadotropins.

### Comparison of the Basic Information and Cycle Status of Infertile Women With Different AMH Levels

AMH levels were classified into low (<2.74), medium (2.74–5.66) and high (≥5.66) according to tertiles. Age (woman and man), basic LH, the number of oocytes obtained, the total amount of Gn, the number of embryos transferred, the number of MII oocytes, basic FSH, basic PRL, and the thickness of the intima were significantly different among the three AMH groups. As shown in **Table 6**, the female and male patients in the high AMH group were the youngest, 29.40 ± 4.0 and 31.6 ± 4.8 years old, respectively, while the patients in the low AMH group were the oldest, 32.60 ± 4.5 and 34.9 ± 6.5 years old, respectively. The high AMH group had a lower basal FSH level of 8.2 ± 2.5 mIU/ml, while the low AMH level group had the highest basal LH level of 9.9 ± 4.1 mIU/ml. LH and the number of oocytes obtained, the number of embryos transferred and the number of MII oocytes of the high AMH group were higher. PRL of the middle-level AMH group was the highest, 16.52 ± 16.38. The Gn dosage of the high AMH group was the lowest, 1998.0 ± 726.9 (**Table 6**).

**Table 6:** Comparison of data of different AMH levels.

Clinical parameters		AMH < 2.74	2.74 ≤ AMH < 5.66	AMH ≥ 5.66	*P*
		(n = 104)	(n = 105)	(n = 105)	
The age of women		32.6 ± 4.5	31.0 ± 4.4	29.4 ± 4.0	<0.001
The age of men		34.9 ± 6.5	32.8 ± 5.5	31.6 ± 4.8	<0.001
Years of infertility		4.6 ± 3.9	4.7 ± 3.7	3.9 ± 2.7	0.197
BMI(kg/m^2^)		22.6 ± 3.3	22.0 ± 4.2	23.1 ± 5.1	0.178
Basic FSH(mIU/ml )		9.9 ± 4.1	9.1 ± 3.3	8.2 ± 2.5	0.001
LH(mIU/ml )		3.2 ± 1.9	3.5 ± 2.3	5.8 ± 6.4	<0.001
Basic E_2_(pg/ml )		142.3 ± 367.6	261.6 ± 622.1	143.8 ± 380.9	0.112
PRL		13.0 ± 6.0	16.52 ± 16.38	13.5 ± 8.3	0.048
Basic T		50.1 ± 26.2	46.5 ± 23.1	51.9 ± 20.0	0.227
Basic P		1.3 ± 3.3	2.0 ± 5.0	1.9 ± 4.4	0.495
AMH(ng/ml )		1.5 ± 0.7	4.0 ± 0.8	10.3 ± 3.7	<0.001
Intimal thickness(mm)		7.0 ± 2.9	6.5 ± 2.7	5.9 ± 2.4	0.017
Number of eggs obtained (pieces)		6.9 ± 3.9	9.9 ± 4.0	11.1 ± 4.4	<0.001
The total of Gn		3088.8 ± 969.4	2840.0 ± 865.7	1998.0 ± 726.9	<0.001
The total days of Gn		11.1 ± 2.5	11.1 ± 1.9	11.2 ± 1.9	0.949
Number of embryos transferred (pieces)		1.7 ± 0.5	1.7 ± 0.5	1.8 ± 0.5	<0.001
Number of MII (pieces)		6.2 ± 3.7	9.1 ± 4.0	10.2 ± 4.3	<0.001
Rate of obtaining mature eggs %		89.3 ± 0.2	90.6 ± 0.1	92.2 ± 0.1	0.343
Ending	Clinical pregnancy	36(34.6%)	29(27.6%)	40(38.1%)	0.083
	Biochemical pregnancy	8(7.7%)	16(15.2%)	16(15.2%)	
	Not pregnant	55(52.9%)	58(55.2%)	49(46.7%)	

BMI, body mass index; FSH, follicle stimulating hormone; LH, luteinizing hormone E_2_, estradiol; AMH, Anti-Mullerian hormone; Gn, gonadotropins.

### Multivariate Logistic Regression Analysis

Statistically significant variables were included in the multivariate logistic regression model. The results showed that age (OR = 0.574 95% CI: 0.350–0.940, *p* = 0.027), AMH (OR = 1.430 95% CI: 1.130–1.820, *p* = 0.003) and the number of oocytes obtained (OR = 1.360 95% CI: 1.030–1.790, *p* = 0.032) were independent factors influencing clinical pregnancy (**Table 7**).

**Table 7:** Logistic regression analysis of clinical pregnancy and abortion factors.

Clinical parameters	*OR*	95% *CI*	*P*
The age of women	0.574	0.350–0.940	0.027
The age of men	1.007	0.945–1.073	0.827
Basic FSH (mIU/ml )	1.042	0.967–1.124	0.278
Basic LH (mIU/ml )	0.987	0.928–1.050	0.681
Basic PRL	1.003	0.983–1.025	0.749
AMH(ng/ml )	1.43	1.130–1.820	0.003
Intimal thickness (mm)	1.065	0.973–1.165	0.173
Number of eggs obtained (pieces)	1.36	1.030–1.790	0.032
The total of Gn	1	1.000–1.000	0.731
Number of embryos transferred (pieces)	1.404	0.840–2.348	0.195
Number of MII (pieces)	1	0.846–1.182	0.999

BMI, body mass index; FSH, follicle stimulating hormone; LH, luteinizing hormone E_2_, estradiol; AMH, Anti-Mullerian hormone; Gn, gonadotropins.

## Discussion

In recent years, due to low ovarian reserve, infertile women have faced problems such as low pregnancy rate and live birth rate, which has become a major challenge for reproductive physicians. The number and quality of women’s oocytes decrease with age but the fertility of women of the same age varies greatly, hence, the accuracy of predicting pregnancy outcome by age only is low. The level of AMH remains relatively constant during the menstrual cycle and can reflect ovarian reserve. Therefore, the application of AMH in advanced-age ARTs has become one of the research hotspots among local and international scholars. A total of 314 advanced-age infertile women were included in this study and the AMH variation range of different age groups of infertile women was established. The correlation between AMH and the number of ovulation-inducing women in different age groups was analyzed, and the independent influence of clinical pregnancy was explored. This should help to guide elderly infertile women seeking IVF treatment.

Because of the lack of a standardized method to measure AMH levels and variation in AMH levels caused by different methods (6, 14), it is necessary to establish a reference range for AMH for different age groups. The patients included in this study were aged 22–43 years old, with an average age of 31.0 ± 4.5 years. The patients were divided into three groups according to their ages. The levels of AMH of patients of different age groups were described and compared. It was found that the average AMH level increased with age. The levels of AMH exhibited a decreasing trend and varied widely in each age group. This is consistent with the results of previous studies (15, 16). AMH is considered the most reliable indicator of ovarian reserve. AMH can reflect the number of antral follicles and is directly proportional to the number of oocytes obtained in hyperstimulation (17, 18). Studies have shown that patients with the same AMH level of different ages have a lower number of ovulation-stimulation than patients in the elderly group, which indicates that age is one of the key factors in the study of ovarian stimulation effects (19). This study compared the correlation between the level of AMH and the number of oocytes obtained in infertile women of different ages and found that the level of AMH in infertile women was positively correlated with the number of oocytes obtained. A further comparison of the levels of AMH in different age groups showed that the levels of AMH in women aged 29–43 were linearly correlated with the number of oocytes harvested, with a statistical significance, which is consistent with findings from previous research (20–22).

We also compared the pregnant and the non-pregnant groups and found that there were statistical differences in the age, the number of oocytes retrieved and the number of embryos transferred between the two groups, suggesting that these three variables could be factors affecting clinical pregnancy.

After classifying the levels of AMH in advanced-age infertile women into three groups (low, medium and high), we found that age, basic LH, number of oocytes harvested, total Gn dosage, number of embryos transferred, number of MII oocytes, basic FSH, basic PRL and intimal thickness were significantly different among the groups. However, our findings are different from a German study (8) perhaps due to the age of the included population; age affects the outcome of assisted pregnancy.

A European study of women over 40 years of age found that compared with the non-pregnant group, patients in the pregnant group had lower ages and higher levels of AFC and AMH, but considered that AMH was related to those over 40 years of age.

The pregnancy outcome in women based on IVF-ET is not relevant (23). Some researchers believe that AMH can predict the clinical pregnancy of IVF in elderly women. Lee analyzed the relationship between the level of AMH and clinical pregnancy in women older than 40 years and reported that AMH is an independent factor that can predict pregnancy (24). The present study further compared the clinical data of the pregnant and the non-pregnant groups and the data of patients with different AMH levels. There were differences in factors such as age, AMH and the number of oocytes obtained between the groups. After incorporating them into the multivariate logistic regression model, age, AMH and the number of oocytes obtained were independent factors in clinical pregnancy. Two European and American studies also found that age and AMH were independent factors predicting IVF pregnancy outcome, which is consistent with our results (25, 26).

This study had some strengths and weaknesses. First, to reduce selection bias, this study only included patients in the first IVF cycle. In addition, patients were further grouped by age to reduce the heterogeneity caused by age changes and the relationship between the number of oocytes obtained and AMH was analyzed by age. However, this study had a small sample size and is a retrospective study with many confounding factors. Furthermore, some variables were not considered in the study.

## Conclusions

We found that the level of AMH in infertile women decreased with age and the number of oocytes obtained in infertile women aged 29–43 was positively correlated with AMH. Age, AMH and the number of oocytes obtained were independent factors influencing clinical pregnancy. Moreover, the number of oocytes and embryo transferred in the pregnant group was significantly higher than that in the non-pregnant group. Furthermore, the clinical pregnancy was affected by age, AMH and number of oocytes. To the best of our knowledge, this is the first study to retrospectively analyze the correlation between AMH and the number of oocytes obtained by COH in women of different ages. This information could help understand the relationship between AMH and IVF-ET IN clinical pregnancy.

## Data Availability Statement

The raw data supporting the conclusions of this article will be made available by the authors, without undue reservation.

## Ethics Statement

The studies involving human participants were reviewed and approved by Ethics Committee of Affiliated Hospital of Southwest Medical University. Written informed consent for participation was not required for this study in accordance with the national legislation and the institutional requirements.

## Author Contributions

XS and YL conceived and designed the study. SL and XL collected patient data, XM and LL reviewed and edited the manuscript. All authors contributed to the article and approved the submitted version.

## Funding

The study was supported by the project of Luzhou science and Technology Bureau (no. 16188) and Science Foundation of Southwest Medical University (no. 2016217).

## Conflict of Interest

The authors declare that the research was conducted in the absence of any commercial or financial relationships that could be construed as a potential conflict of interest.
